# Identification of pyrrolopyrimidine derivative PP-13 as a novel microtubule-destabilizing agent with promising anticancer properties

**DOI:** 10.1038/s41598-017-09491-9

**Published:** 2017-08-31

**Authors:** Pauline Gilson, Fernando Josa-Prado, Claire Beauvineau, Delphine Naud-Martin, Laetitia Vanwonterghem, Florence Mahuteau-Betzer, Alexis Moreno, Pierre Falson, Laurence Lafanechère, Véronique Frachet, Jean-Luc Coll, Jose Fernando Díaz, Amandine Hurbin, Benoit Busser

**Affiliations:** 1 Cancer Target and Experimental Therapeutics, Institute for Advanced Biosciences, INSERM U1209, CNRS UMR5309, Grenoble Alpes University, Grenoble, France; 2 Biochemistry, Toxicology and Pharmacology Department, Grenoble University Hospital, Grenoble, France; 3 Centro de Investigaciones Biológicas, Consejo Superior de Investigaciones Científicas, Madrid, Spain; 4 Institut Curie, PSL Research University, CNRS, INSERM UMR9187/U1196, Orsay, France; 5 Drug Resistance Mechanisms and Membrane Proteins Laboratory, MMSB UMR 5086 CNRS/ Lyon 1 University, Institute of Biology and Chemistry of Proteins, Lyon, France; 6 EPHE, PSL Research University, Paris, France

## Abstract

Despite the emergence of targeted therapies and immunotherapy, chemotherapy remains the gold-standard for the treatment of most patients with solid malignancies. Spindle poisons that interfere with microtubule dynamics are commonly used in chemotherapy drug combinations. However, their troublesome side effects and the emergence of chemoresistance highlight the need for identifying alternative agents. We performed a high throughput cell-based screening and selected a pyrrolopyrimidine molecule (named PP-13). In the present study, we evaluated its anticancer properties *in vitro* and *in vivo*. We showed that PP-13 exerted cytotoxic effects on various cancer cells, including those resistant to current targeted therapies and chemotherapies. PP-13 induced a transient mitotic blockade by interfering with both mitotic spindle organization and microtubule dynamics and finally led to mitotic slippage, aneuploidy and direct apoptotic death. PP-13 was identified as a microtubule-targeting agent that binds directly to the colchicine site in β-tubulin. Interestingly, PP-13 overcame the multidrug-resistant cancer cell phenotype and significantly reduced tumour growth and metastatic invasiveness without any noticeable toxicity for the chicken embryo *in vivo*. Overall, PP-13 appears to be a novel synthetic microtubule inhibitor with interesting anticancer properties and could be further investigated as a potent alternative for the management of malignancies including chemoresistant ones.

## Introduction

In the last two decades, advances in the understanding of carcinogenesis have revolutionized the management of cancer patients with the development of targeted therapies and immunotherapy. Identification of genetic alterations in subsets of cancers leading to different subcellular signals of tumour growth and progression elicited the clinical use of oncogene-targeted therapies for patients harbouring such anomalies^1–4^. In lung cancers with EGFR-activating mutations, anti-EGFR therapies, including gefitinib, erlotinib, and afatinib, have been shown to improve progression-free survival and were approved as first-line options^5^. However, despite the initial response, resistance mechanisms almost inevitably ensue and limit the long-term potency of targeted therapies^6, 7^. Moreover, efforts are still needed for the management of patients without targetable oncogenic driver mutations representing 60% of non-small cell lung cancer (NSCLC) cases^8^. Immunotherapies, especially immune checkpoint inhibitors, are emerging for cancer therapy and have the advantage of providing durable responses and improved patient prognosis^9–11^; however, intrinsic resistance for numerous tumour types has also restricted their use^12^. Therefore, non-selective chemotherapy remains a gold-standard treatment for almost all patients with advanced solid cancers. Spindle poisons that interfere with microtubule dynamics represent a major class of conventional chemotherapy drugs commonly used in combination for cancer treatments^8^. Among the approved microtubule-targeting agents, taxanes and vinca-alkaloids that respectively stabilize and depolymerize microtubules are widely used. However, their troublesome side effects, especially myelosuppression and peripheral neurotoxicity, and the emergence of chemoresistance make essential the development of alternative agents^13^.

With the aim to identify new chemical compounds able to abolish resistance to apoptosis in NSCLC, we have conducted a high-throughput cell-based screening of more than 7500 molecules. We have selected a small molecule belonging to the pyrrolopyrimidine family (PP-13) that strongly induced apoptosis. Here, we show that PP-13 is cytotoxic for a large panel of human cancer cell lines. The PP-13 molecule induced mitotic cell blockade by impairing both mitotic microtubule organization and dynamics and was identified as a microtubule-targeting agent that selectively binds to the colchicine-binding site on β-tubulin. Contrary to conventional spindle poisons used for cancer treatment, PP-13 overcame multidrug resistance (MDR). This molecule also showed *in vivo* antitumour activity without any noticeable toxicity in the chicken embryo.

## Results

### Identification of a new molecule inhibiting the proliferation of various human cancer cells

**Figure 1:**
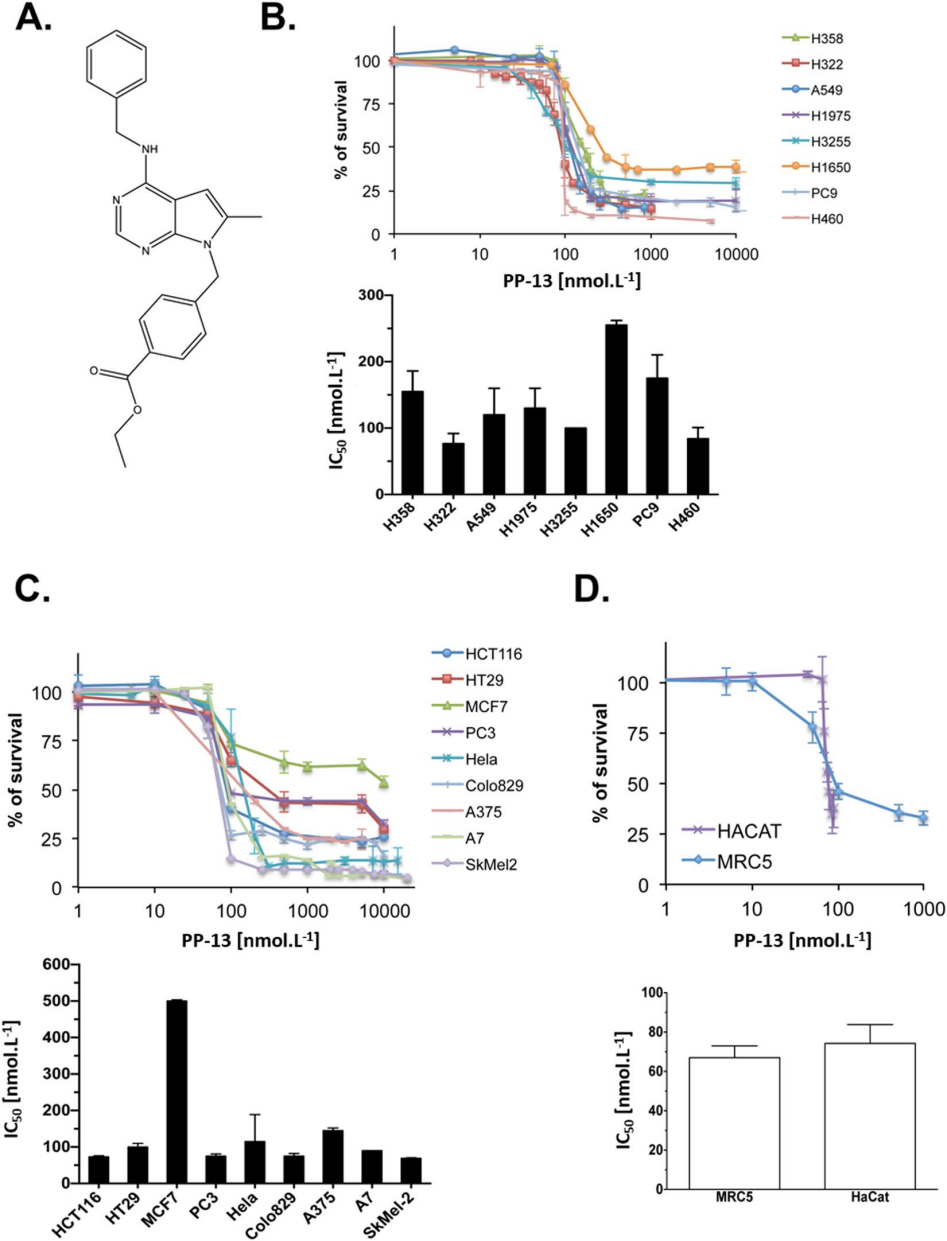
PP-13 significantly inhibited the proliferation of human cancer cell lines. (**A**) Chemical structure of PP-13. (**B–D**) The MTT assays in NSCLC cells (**B**), in other representative cancer cell lines from various origins (**C**), and in human foetal lung fibroblast MRC5 cells and in human keratinocyte HaCat cells (**D**), treated with the indicated concentrations of PP-13 for 72 h. Lower panels: PP-13 concentrations required to inhibit cell growth by 50% (IC_50_) at 72 h. Data represent the mean ± SD of three independent experiments (in nmol.L^−1^).

To identify new potent chemical molecules that induce apoptosis in a resistant NSCLC cell model, we designed a high-throughput cell-based assay on H358 NSCLC cells that we have previously described as a model of resistance to apoptosis induced by serum starvation^14, 15^. We screened 7520 compounds at a final concentration of 2.5 µmol.L^−1^. Among the 71 chemical molecules identified as restoring more than 49% of apoptosis, one pyrrolopyrimidine derivative, PP-13 (ethyl 4-((4-(benzylamino)-6-methyl-7H-pyrrolo[2,3-d]pyrimidin-7-yl)methyl)benzoate), was finally selected for further biological and biochemical characterization owing to its high cytotoxic effects (Fig. 1A).

We first evaluated the ability of PP-13 to inhibit growth of human NSCLC cell lines (H358, H322, A549, H1975, H3255, H1650, PC9 and NCI-H460) harbouring various forms of *TP53*, *KRAS* and *EGFR* status (Supplementary Fig. S1). NSCLC cells treated with increasing concentrations of PP-13 showed a drastic inhibition of their viability regardless of their mutational status (Fig. 1B upper panel). Concentration values inhibiting cell growth by 50% (IC_50_) ranged from 76 to 255 nmol.L^−1^ (Fig. 1B lower panel). Interestingly, PP-13 was effective both on NSCLC cell lines resistant (H1650, H1975) and sensitive (PC9, H3255) to anti-EGFR-targeted therapies. To determine if PP-13 activity was specific to NSCLC cells, we used other representative human cancer cell lines from various origins (colorectal cancer cell lines HCT116 and HT29; breast cancer cell line MCF7; prostate cancer cell line PC3; cervical cancer cell line HeLa; melanoma cell lines colo829, A375, A7 and SkMel-2) (Fig. 1C). Similar to the results obtained in NSCLC cells, the IC_50_ concentrations for PP-13 ranged from 67 to 145 nmol.L^-1^, except for MCF7 cells, which resisted to PP-13. PP-13 also reduced the viability of normal human foetal lung fibroblasts, MRC5, and human keratinocyte, HaCat, with an IC_50_ of about 70 nmol.L^-1^ in the same range as for cancer cell lines (Fig. 1D). Similar effects were observed in these cell lines with the antimitotic chemotherapy paclitaxel currently used for breast cancers, ovarian cancers, or NSCLC treatment (Supplementary Fig. S2). Although IC_50_ concentrations for PP-13 were higher than those for paclitaxel in cancer cell lines, they were in the nanomolar range (Fig. 1 and Supplementary Fig. S2). In addition, MRC5 and HaCat normal cells appeared to be less sensitive to PP-13 compared to paclitaxel (Fig. 1D and Supplementary Fig. S2C). Taken together, these data suggest that PP-13 exerts an interesting cytotoxic activity in a wide panel of cancer cell lines.

### PP-13 overcomes the multidrug-resistant (MDR) phenotype in cancer cells

The overexpression of efflux pumps or multidrug transporters confers cell resistance to many drugs and represents the major explanation for the mechanism of tumour cell chemoresistance to spindle poisons^16^. To determine the activity of PP-13 in an MDR phenotype context, we compared the effects of PP-13 on the proliferation of drug-sensitive cells with those on their drug-resistant counterparts that overexpress P-glycoprotein, BCRP, MRP1, or MRP2 efflux transporters (Table 1). PP-13 exerted similar cytotoxic effects in drug-sensitive cells and MDR cells, with an IC_50_ ranging between 280 nmol.L^−1^ and 1 µmol.L^−1^. This result indicates that PP-13 is not a substrate of these drug transporters. This contrasts with the active efflux of paclitaxel by P-glycoprotein, with a ratio of 375 between the IC_50_ of drug-sensitive and P-glycoprotein-overexpressing cells (Table 1 and ref. 16).

**Table 1:** PP-13 overcomes efflux-mediated chemoresistance. The effects of PP-13 and paclitaxel on cell viability were determined by MTT assays. Concentrations required to inhibit cell growth by 50% (IC_50_) at 72 h in drug-sensitive cell lines and their multidrug-resistant counterparts overexpressing P-glycoprotein, MRP1, MRP2, or BCRP transporters. Data are the mean of triplicates. nt: not tested.

Cell lines	IC_50_ of PP-13	IC_50_ of Paclitaxel
HEK293	780 nmol.L^−1^	nt
HEK293 overexpressing MRP2 transporter	700 nmol.L^−1^	nt
NIH-3T3	490 nmol.L^−1^	40 nmol.L^−1^
NIH-3T3 overexpressing P-glycoprotein transporter	490 nmol.L^−1^	>15 µmol.L^−1^
HEK293	1000 nmol.L^−1^	5 nmol.L^−1^
HEK293 overexpressing MRP1 transporter	650 nmol.L^−1^	6.3 nmol.L^−1^
HEK293	430 nmol.L^−1^	75 nmol.L^−1^
HEK293 overexpressing BCRP transporter	280 nmol.L^−1^	93 nmol.L^−1^

### PP-13 induces mitotic blockade in cancer cells

**Figure 2:**
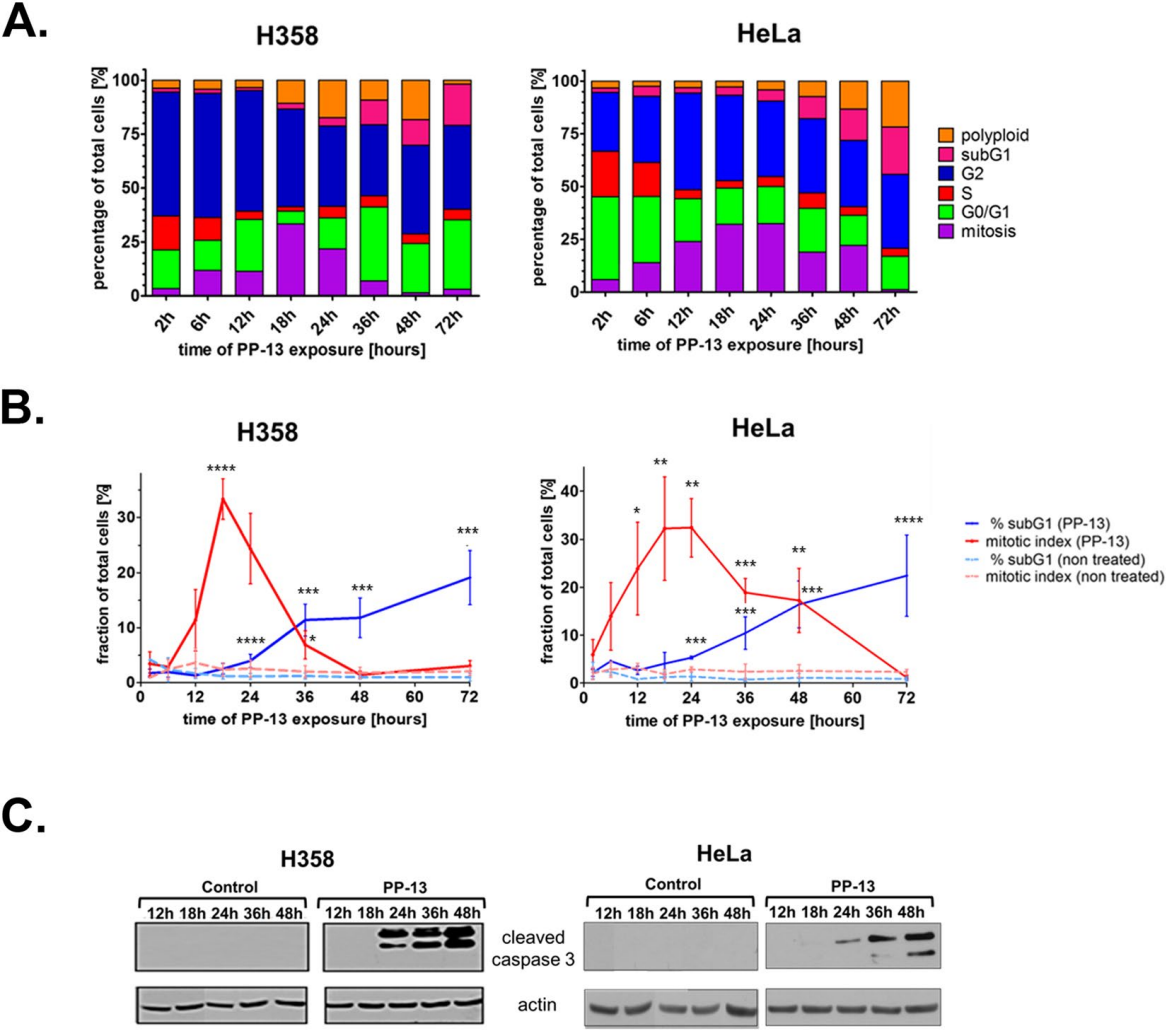
PP-13 interferes with the mitotic progression of H358 NSCLC and HeLa cells. H358 and HeLa cells were treated with PP-13 at 170 and 120 nmol.L^−1^, respectively. (**A**) The cell-cycle phases were determined by flow cytometry over time as indicated. The histograms represent the mean of the percentage of cells in each phase of the cell cycle (n = 3 independent experiments). (**B**) Graphical representation of the percentage of mitotic and sub-G_1_ cells in control and PP-13-treated cells. Data are presented as the mean ± SD of ≥ 3 independent experiments (*****p < 0.01; ****0.01 < p < 0.02; ***0.02 < p < 0.03; **0.03 < p < 0.04, *0.04 < p < 0.05 compared to control cells). (**C**) Cleavage of caspase 3 was evaluated by western blotting. Actin was used as a loading control. For the sake of clarity, cropped blots are shown. Full-length blots are presented in Supplementary Fig. S4.

Two cell lines presenting high sensitivity to PP-13 were chosen for further investigation: the H358 NSCLC cells that were used for the initial high-throughput cell-based screening model and the HeLa carcinoma cell line, with IC_50_ values of 170 and 120 nmol.L^−1^, respectively. The effects of PP-13 on cell cycle distribution were assessed over time by flow cytometry in these cells. Both H358 and HeLa cells treated with PP-13 at the IC_50_ concentration transiently accumulated in the mitosis stage (Fig. 2A,B). Thirty-two percent of cells were found in mitosis after 18 h of PP-13 exposure compared to approximately 2% of vehicle-treated cells (Fig. 2A,B, and Supplementary Fig. S3). The percentage of mitotic cells then gradually decreased, concomitantly with the accumulation of apoptotic cells in the sub-G_1_ fraction. After 72 h of PP-13 treatment, approximately 20% of cells underwent apoptosis compared to only 1% of the vehicle-treated cells. Consistent with the progressive increase in the apoptotic sub-G1 cells, PP-13 strongly induced the cleavage of caspase-3 in both H358 and HeLa cells (Fig. 2C and Supplementary Fig. S4). Although PP-13 effect was not as strong as that of paclitaxel^17^ (Supplementary Fig. S2), these results showed that PP-13 induced mitotic cell arrest, which ultimately led to caspase-dependent apoptosis.

### PP-13 interferes with both mitotic microtubule organization and spindle pole integrity

**Figure 3:**
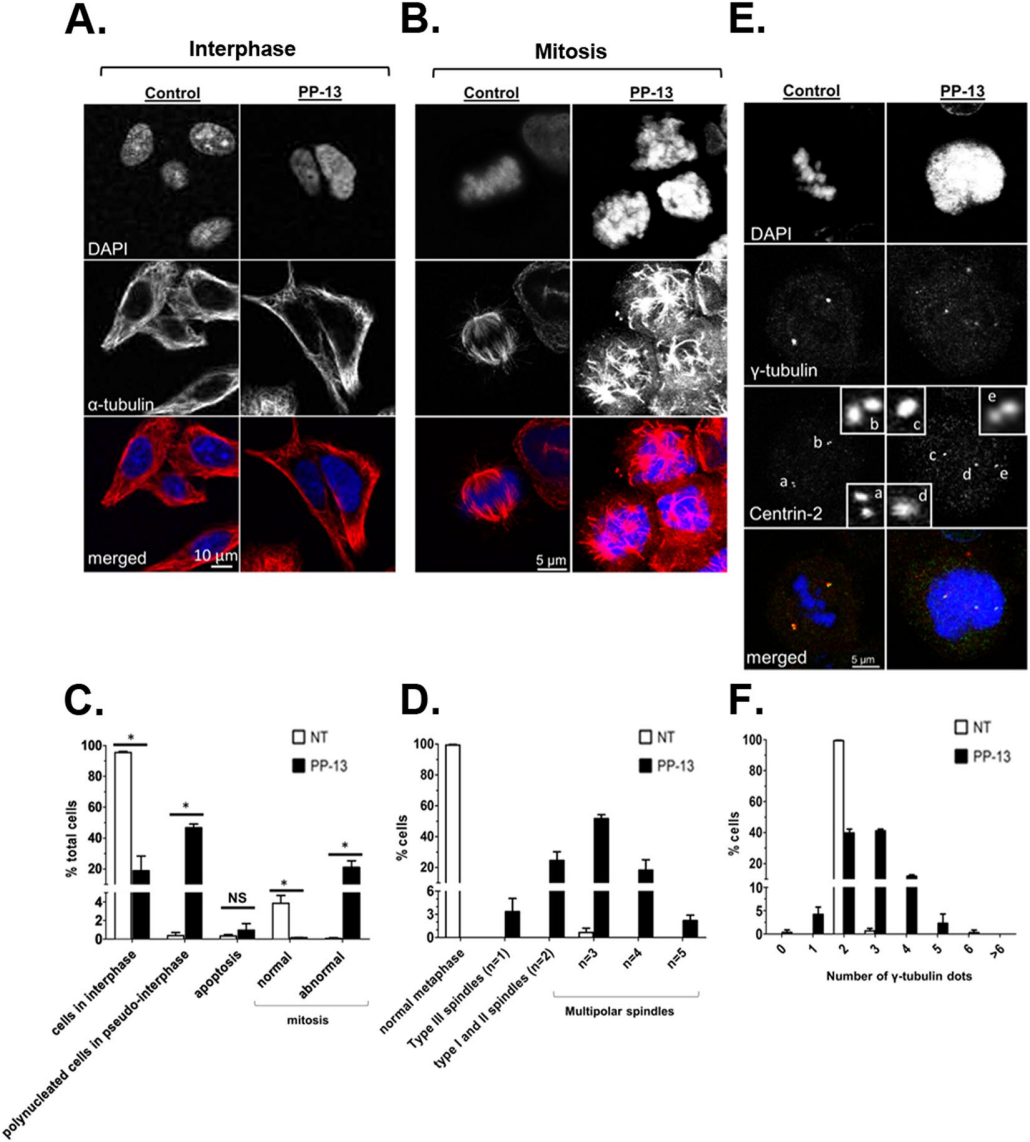
PP-13 interferes with mitotic spindle architecture and centrosome formation. HeLa cells were treated with or without 120 nmol.L^−1^ PP-13 for 24 h. (**A**,**B**) Representative confocal microscopy images of microtubules in cells in interphase (**A**) or mitosis (**B**). In red: α-tubulin, in blue: DNA staining with DAPI. (**C**) Quantification of interphase, mitotic, apoptotic and polynucleated cells. At least 550 cells were randomly chosen and scored in each condition. *p = 0.0495; NS, not significant. (**D**) Quantification of spindle poles per mitotic cell. According to a conventional nomenclature^60^, type I and II spindles are bipolar (n = 2) with at least one uncongressed chromosome; type III spindles are monopolar (n = 1), whereas multipolar spindles presented more than two poles (n > 2). At least 100 mitotic cells were randomly chosen and scored in each condition. n: number of mitotic spindles. (**E**) Confocal microscopy images showing centrosome organization and centrin-2 repartition in mitotic cells. Insets represent a zoom of the boxed area. In red: γ-tubulin, in green: centrin-2, in blue: DNA staining with DAPI, a-e: centrioles. (**F**) Quantification of γ-tubulin dots per mitotic cell. At least 100 mitotic cells were randomly chosen and scored in each condition.

Since any perturbation of the microtubule dynamic balance can lead to microtubule disorganization and mitotic cell cycle arrest^18^, we investigated the effects of PP-13 on microtubules in HeLa cells by immunofluorescence. After a 24h treatment with PP-13, HeLa cells presented a microtubule organization in interphase similar to that of control cells (Fig. 3A). During metaphase, control cells typically showed 2 symmetrical and fusiform bipolar spindles with well-aligned chromosomes at the mid-plate (Fig. 3B). In contrast, PP-13-driven mitotically arrested cells showed short and multipolar spindles associated with strong defects in chromosome congression and alignment, suggesting a prometaphase blockade. In agreement with the flow cytometry analysis, the percentage of cells in interphase decreased in response to PP-13 treatment, whereas abnormal mitotic cells accumulated (Fig. 3C). Moreover, huge and polynucleated cells in pseudo-interphase were also identified and accounted for almost 47% of total cells. Abnormal mitotic cells showed a mean of 2–4 spindle poles (Fig. 3D). Similar observations were made in PP-13-treated H358 cells, but the cells mostly showed more than 5 spindles poles and no significant augmentation of polynucleated cells (Supplementary Fig. S5). Since aberrant multipolar spindles are frequently associated with supernumerary centrosomes^19^, we quantified the microtubule-organizing centres (MTOCs) in mitotic cells using γ-tubulin staining (Fig. 3E and F). Under PP-13 treatment, HeLa cells displayed a variable number of γ-tubulin dots, with an average of 2–3 γ-tubulin dots per cell, while control cells normally harboured 2 γ-tubulin dots. In contrast, in H358 cells, the number of γ-tubulin dots was comparable to that of control cells (Supplementary Fig. S5). We next examined the centrosome composition with centrin-2, a specific marker for centrioles. Control cells showed each centrosome displaying a pair of cohesive centrioles (Fig. 3E), whereas PP-13-treated cells with more than 2 spindle poles presented variable numbers of centrin-2 dots (0, 1 or 2 dots per MTOC), likely because of the disengagement of centriole pairs^20^. Overall, these observations showed that PP-13 induced mitotic spindle multipolarity and microtubule disorganization, resulting in prometaphase cell cycle arrest.

### PP-13 activates the spindle assembly checkpoint followed by mitotic slippage, aneuploidy or direct apoptotic death

**Figure 4:**
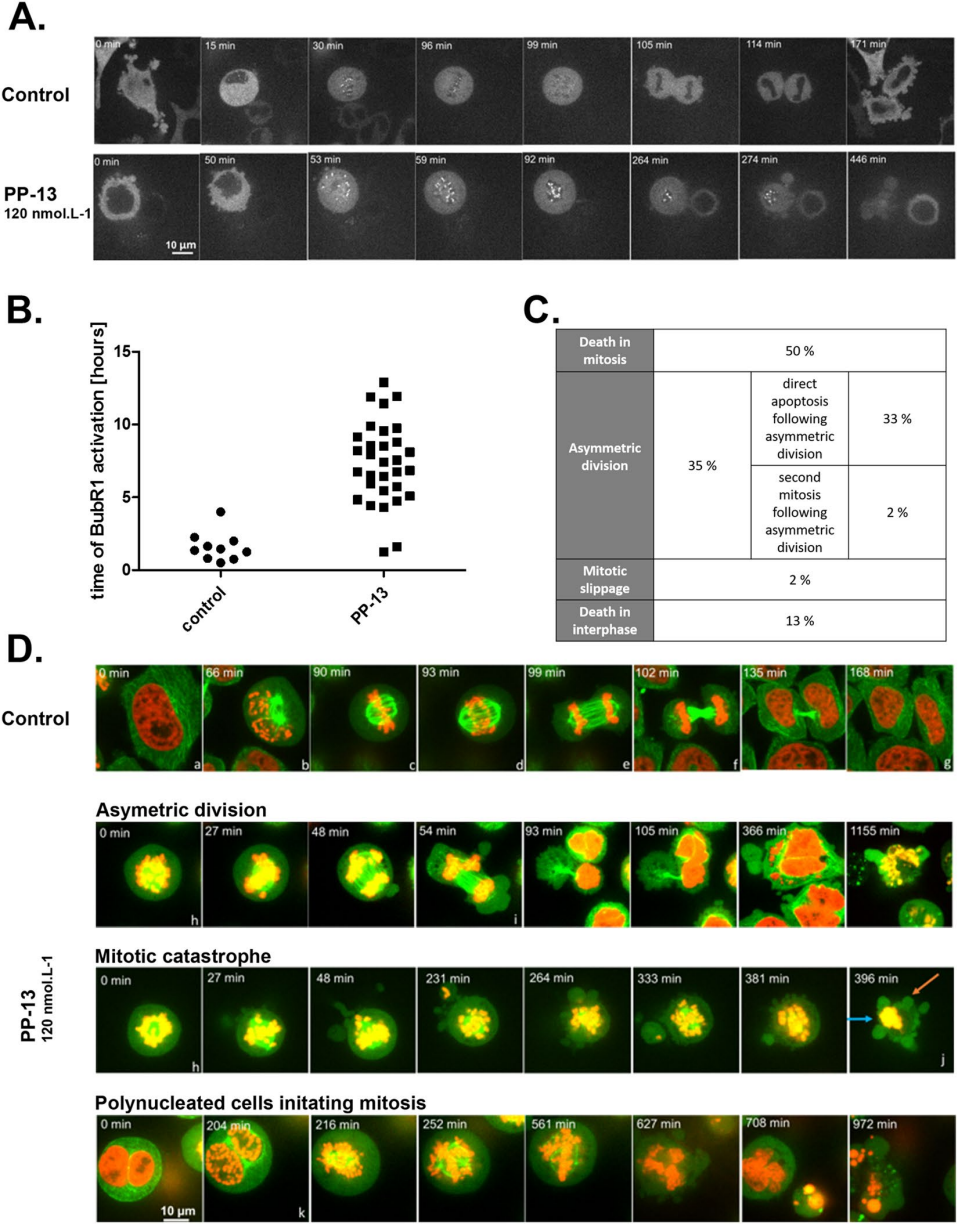
PP-13 induces cell prometaphase arrest then asymmetric division or direct apoptotic death. BubR1-EGFP HeLa cells (**A**,**B**) or α-tubulin-EGFP H2B-RFP HeLa cells (**C**,**D**) were treated with or without 120 nmol.L^−1^ PP-13, and cells were followed over time with time-lapse videomicroscopy. Times are indicated on each picture. (**A**) Spindle assembly checkpoint (SAC) activation was analysed over time by following the activation of BubR1. (**B**) Time of BubR1 activation was estimated by time-lapse videomicroscopy, after following 10 control cells and 31 treated cells over time. Only 2/31 treated cells had BubR1 activation time less than 2 hours due to mitotic catastrophe. (**C**) Percentages of different cell fates induced by PP-13 treatment were estimated by time-lapse videomicroscopy, after following 46 treated cells over time. (**D**) The cell division process was followed over time and representative pictures are shown. Control cells undergoing mitosis exhibit typical features of the different cell-cycle phases: a. interphase; b. prophase; c. metaphase; d. anaphase I; e. anaphase II; f. cytokinesis; g. interphase. PP-13 induces cell prometaphase arrest (h), then asymmetric division (i) or direct apoptotic death (j). Orange and blue arrows indicate blebbing and chromatin condensation respectively. Polynucleated cells generated from asymmetric division can initiate a second mitosis (k). In red: H2B-RFP, in green: α-tubulin-EGFP.

The spindle assembly checkpoint (SAC) is a safety mechanism assuring faithful chromosome segregation during cell division. The SAC is typically activated during prometaphase and delays anaphase onset until all chromosomes are well aligned and correctly attached to the mitotic spindle by kinetochores. Since the SAC complex is frequently involved in prometaphase arrest^21^, we evaluated the impact of PP-13 on SAC activation by following the activation of BubR1, a major component of SAC (Fig. 4A and B). Time-lapse videomicroscopy of HeLa cells stably expressing BubR1-EGFP showed that the mean time of BubR1 activation in control cells was 1.60 ± 1.01 h, whereas it was more than 7.37 ± 2.79 h in PP-13-treated cells (Fig. 4A and B). Activation of BubR1 was thus approximately 5-times longer in PP-13-treated cells than in control cells, probably contributing to mitotic arrest. The fate of transiently arrested cells was next investigated by time-lapse videomicroscopy using HeLa cells stably expressing α-tubulin-EGFP and histone H2B-RFP (Fig. 4C and D). In control conditions, mitotic cells represented only 2% of cells and exhibited typical mitotic features (Fig. 4D and Supplementary Fig. S6). Under PP-13 treatment, different fates of abnormal mitotic cells were observed. Fifty percent of mitotic cells underwent apoptosis directly as evidenced by the emergence of membrane blebbing, chromatin condensation or apoptotic bodies (Fig. 4C and D, and Supplementary Fig. S7). Following prometaphase blockade, 35% of cells initiated aberrant cytokinesis with asymmetric cytoplasmic and chromosomal division (Fig. 4C and D, and Supplementary Fig. S8). This resulted in cell aneuploidy with the generation of non-viable cells or re-clustering, leading to the generation of polynucleated pseudo-interphase cells. In turn, cells either underwent cell death (94% of these cases), as indicated by the presence of micronuclei, nucleus hyperfragmentation or blebbing, or moved forward into mitosis (6% of these cases) (Fig. 4C and D, and Supplementary Fig. S9). Finally, a small fraction of cells died during interphase (13%) or slipped out of mitosis (2%). Similar defects were obtained with H358 cells, with the induction of either mitotic slippage (exit out of mitosis without cytokinesis, 64% of cases), asymmetric division (24% of cases), or direct apoptotic death (12% of cases) (Supplementary Fig. S10). Altogether, these data showed that PP-13 induced an SAC-dependent prometaphase blockade leading to aneuploidy, mitotic slippage and cell death.

### PP-13 directly targets tubulin and competes with colchicine for binding

**Figure 5:**
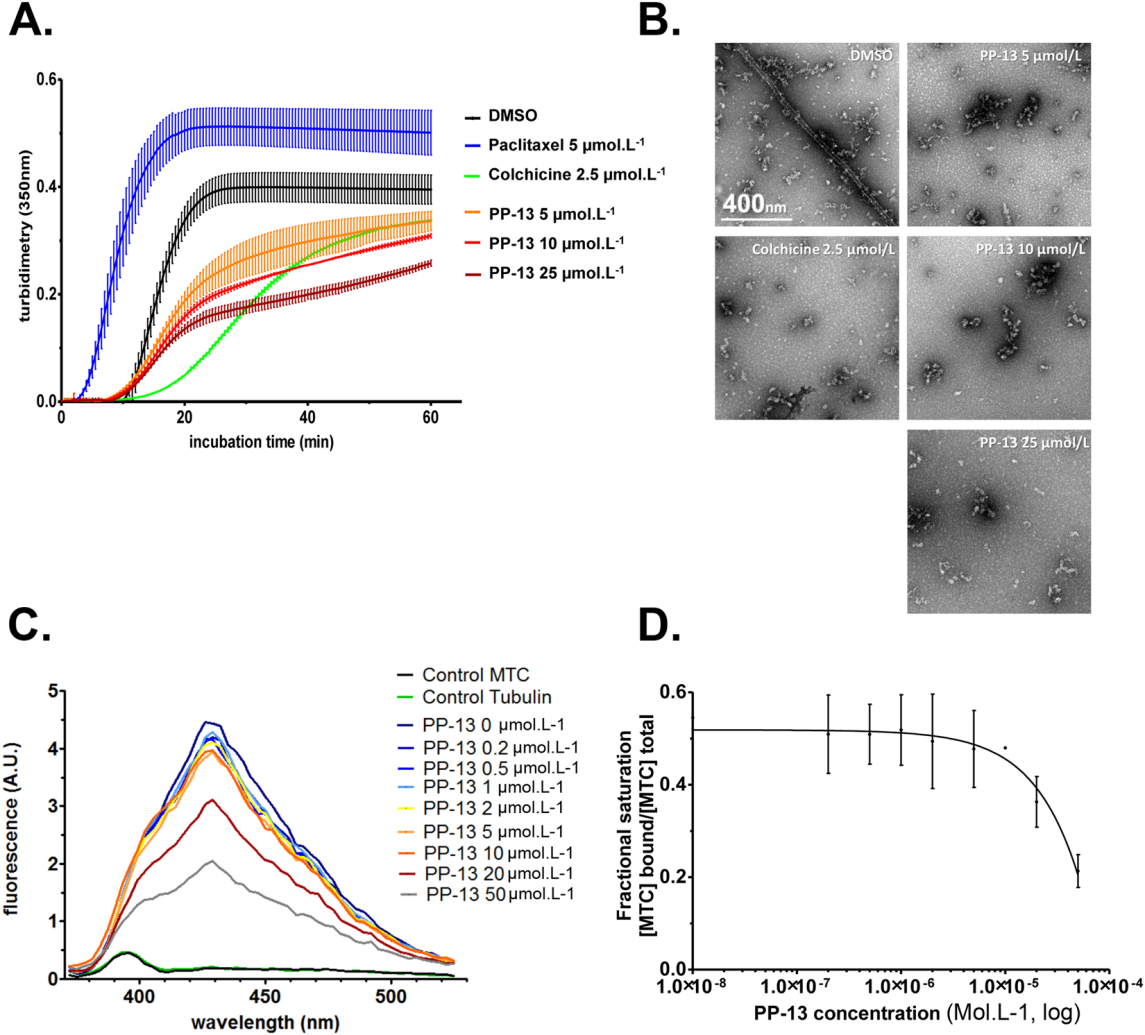
PP-13 is a microtubule-depolymerizing agent that competes with colchicine for tubulin binding. (**A**) Effect of PP-13 on microtubule dynamics assessed by an *in vitro* tubulin polymerization assay over time. Assembly of pure tubulin (40 µmol.L^−1^) in the presence of increasing concentrations of PP-13 (0 to 25 µmol.L^−1^) was followed by turbidimetry at 350 nm. DMSO, paclitaxel (5 µmol.L^−1^) and colchicine (2.5 µmol.L^−1^) were used as vehicle control, polymerizing and depolymerizing agents, respectively. Each turbidity value represents the mean ± SD from 3 independent experiments. (**B**) Representative transmission electron microscopy images showing the polymerization of tubulin (25 µmol.L^−1^) in the presence of DMSO, colchicine (2.5 µmol.L^−1^) or increasing concentrations of PP-13 (0 to 25 µmol.L^−1^). (**C**) Displacement of MTC from the colchicine binding site by PP-13. Fluorescence emission spectra (372–525 nm) of 10 µmol.L^−1^ MTC and 10 µmol.L^−1^ tubulin in the presence of increasing concentrations of PP-13 (0 to 50 µmol.L^−1^) are shown. Each fluorescence intensity value (in arbitrary unit, A.U.) represents the mean from 2 independent assays. Error bars are not shown for the sake of clarity. (**D**) Displacement of MTC from the colchicine-binding ﻿s﻿ite﻿ by PP-13. MTC fractional saturation was calculated based on the concentration of MTC binding sites (0.8 per tubulin dimer) and the MTC binding constant (4.7× 10^5^ M^−1^).

The main molecular targets of PP-13 involved in SAC-dependent prometaphase blockade could be mitotic kinases or tubulin. We first performed a kinome assay to test the ability of PP-13 to inhibit a panel of more than 450 human protein kinases including mitotic kinases and relevant cancer mutant kinases. No kinase was inhibited at 1 or 10 µmol.L^−1^, suggesting that PP-13 does not act as a kinase inhibitor (Supplementary Fig. S11). To evaluate whether tubulin is a target of PP-13, we performed an *in vitro* tubulin polymerization assay using pure tubulin. PP-13 did not delay the start of microtubule assembly but significantly reduced the microtubule polymerization speed and maximal polymerized tubulin rate in a dose-dependent manner (Fig. 5A). After 1 h, 5 µmol.L^−1^ of PP-13 decreased the tubulin polymer mass as much as 2.5 µmol.L^−1^ of colchicine. The microtubule-depolymerizing effect of PP-13 was confirmed by electron microscopy (Fig. 5B). No microtubule was observed in the presence of PP-13 or colchicine. These observations indicated that PP-13 directly targets tubulin and exerts potent depolymerizing effects *in vitro*.

Because the colchicine binding site is a major pocket for most of the microtubule destabilizers^22^, we investigated the ability of PP-13 to bind to this site by competitive experiments with a reversible fluorescent colchicine analogue (MTC) whose binding to unassembled tubulin raises the fluorescence signal^23, 24^. In the conditions of the assay (absence of Mg^+^^2^ in the buffer), tubulin is in the dimeric state. Conditions were chosen to allow that roughly 50% of the colchicine binding sites in tubulin are occupied by MTC and 50% of them are free. In the presence of a competitor for the colchicine site, it will bind to the empty sites and the MTC binding equilibrium will be reshuffled resulting in a decreased intensity of the tubulin-bound MTC fluorescence in a dose-dependent manner, which was observed in Fig. 5C. Fifty µmol.L^−1^ of PP-13 reduced the fluorescence intensity by 56%. This displacement of MTC by PP-13 fits to a competitive model^25^ (Fig. 5D), demonstrating the ability of PP-13 to bind to the colchicine-binding site of tubulin. Based on the MTC binding constant (4.7 × 10^5^ M^−1^), the binding constant of PP-13 was calculated as previously described^25^ to be 9.3 × 10^4^ M^−1^ (Fig. 5D).

### PP-13 reduces tumour growth and metastasis invasion *in vivo*

**Figure 6:**
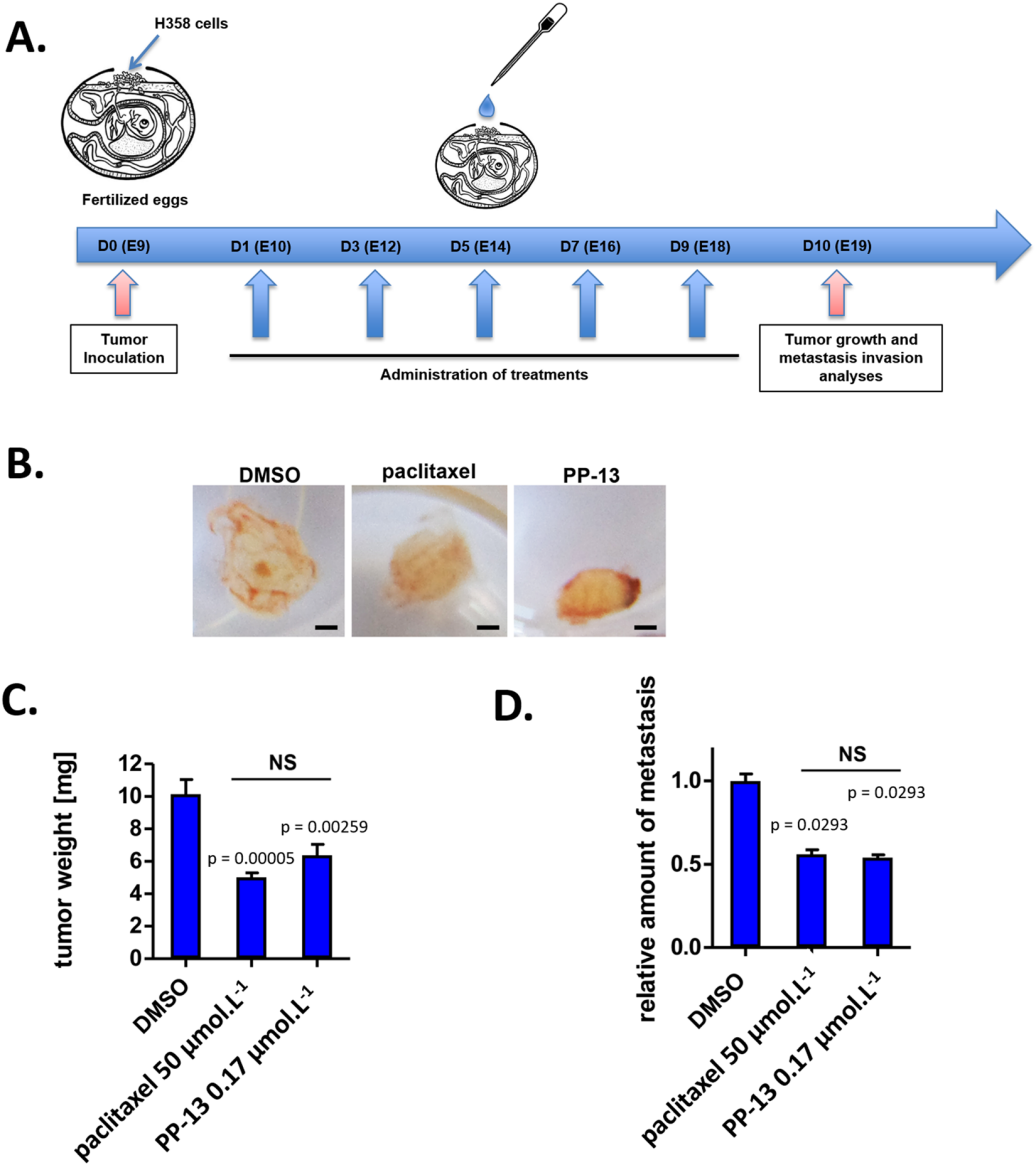
PP-13 inhibits tumour growth and cell dissemination *in vivo*. H358 NSCLC cells were xenografted on a chick embryo chorioallantoic membrane (CAM). After treatment with vehicle (0.5% DMSO), paclitaxel (50 µmol.L^−1^) or PP-13 (170 nmol.L^−1^), tumours were excised and weighed. (**A**) Schematic representation of the assay principle. (**B**) Representatives pictures of tumours at the end of the different treatments. Bar = 1 mm. (**C**) Effects of treatments on the H358 tumour weight (means ± SEM of ≥16 samples). (**D**) Effects of treatments on H358 metastasis in the lower CAM (means ± SEM of 15 samples). The presence of H358 cells in the lower CAM was evaluated by qPCR.

To evaluate the antitumour activity of PP-13 *in vivo*, we used NSCLC H358 cells xenografted on chicken chorioallantoic membranes (CAM). Formed tumours were treated every 48 h with vehicle (0.5% DMSO), paclitaxel (50 µmol.L^−1^), or PP-13 (0.17 µmol.L^−1^) (Fig. 6A). Paclitaxel was used as a positive control and a concentration of 50 µmol.L^−1^ was chosen to induce significant tumour growth inhibition without toxicity for embryos. At day 19, tumours were recovered from the upper CAM and weighed. PP-13 significantly inhibited H358 tumour growth (Fig. 6B and C) compared to control. Importantly, the comparison of the number of dead chicken embryos in control and PP-13-treated eggs, as well as the analysis of embryos morphological or functional defects, indicated that PP-13 showed no noticeable toxicity in the chicken embryo (Supplementary Fig. S12). This suggests that in this model PP-13 is well tolerated at a dose sufficient to induce an anti-tumour effect. Analysis of the presence of H358 cells at the lower CAM by qPCR allowed for the accurate detection of tumour cell dissemination (Fig. 6D). At 0.17 µmol.L^−1^, PP-13 reduced the metastasis ability of H358 cells compared to control, suggesting that PP-13 inhibits invasion mechanisms.

## Discussion

We have isolated a pyrrolopyrimidine derivative (PP-13) that is cytotoxic for several cancer cell lines in the same order of magnitude of concentrations as paclitaxel. MCF7 was the only cell line that resisted to both PP-13 and paclitaxel. One explanation could be the absence of caspase 3 in this cell line^26^. In addition, PP-13 seemed less toxic than paclitaxel for normal cells. In an attempt to decipher its mechanism of action, we carefully analysed its effect on the cell cycle. We found that PP-13 induced spindle multipolarity, chromosome missegregation and subsequent transient mitotic arrest in prometaphase in H358 NSCLC and HeLa cervical cancer cells. Treated cells displayed supernumerary spindle poles likely because of a loss of spindle pole integrity and premature centriole pair disengagement given the atypical composition of MTOCs (0–2 centrioles per pole). A similar aberrant phenotype following mitotic blockade has been described for cells treated with nocodazole, colcemid or nitrous oxide^20^. The consequences of PP-13 treatment on the number of mitotic spindles and spindle poles were found to be highly variable according to the cell type. PP-13-treated HeLa cells exhibited 2–4 mitotic spindles and 2–3 MTOCs, whereas PP-13-treated H358 cells displayed more than 5 mitotic spindles. The number of centrosomes of these latter cells appeared, however, normal, as assessed by γ-tubulin staining, which was equivalent to the staining of the DMSO-treated control cells. This could suggest that either the PP-13 molecular targets or mitotic checkpoints differ depending on the cell type. In addition, PP-13 induced an accumulation of polynucleated cells in the G_1_-like stage in HeLa cells and not in H358 cells. These different mitotic phenotypes observed between the different cell types could be explained by their p53 status, as p53 is a well-known sensor of mitotic failure^27^.

In PP-13-treated cells, spindle multipolarity led to defects in chromosome alignment and spindle-kinetochore attachment. Thus, the lack of tension between the two sister kinetochores did not satisfy the spindle assembly checkpoint (SAC)^28^. This is most likely the reason for the prolonged mitotic arrest observed in live imaging of HeLa cells expressing the major component of the SAC BubR1. Using time-lapse videomicroscopy, we observed that cells finally exited from prolonged mitotic arrest with various cell fates, as described elsewhere^29^. We and others^30, 31^ believe that the cell type might largely influence the cell outcome. We observed that half of HeLa cells died immediately during mitosis *via* mitotic catastrophe as evidenced by the presence of blebs, chromatin condensation and the release of apoptotic bodies, while the other cells (35%) escaped from the mitotic blockade through asymmetric division generating non-viable aneuploid cells. On the other hand, a large fraction of H358 cells (64%) exited mitosis without cell division (mitotic slippage), then died during interphase or a second mitosis.

Analysis of the effect of PP-13 on the kinetics of *in vitro* pure tubulin assembly indicated that PP-13 is able to inhibit tubulin assembly. This was confirmed by electron microscopy analysis of microtubules treated with PP-13. Competition experiments indicated that PP-13 binds tubulin at the colchicine-binding site, with an apparent binding affinity constant (Kb) of 9.3 × 10^4^ M^−1^. The PP-13 Kb value can explain, at least in part, why the effects of PP-13 on microtubule organization are only observed in mitotic cells but not in interphase cells at the IC_50_. The mass action law predicts that only 1% of PP-13 binds to the available binding sites, since its IC_50_ is 100 times less than its dissociation constant (1/Kb). Since tubulin disassembly by colchicinoids is stoichiometric, PP-13 would not induce a visible effect in the polymer mass at IC_50_ concentrations during interphase. Since most neurons mainly remain in interphase, PP-13 could have a reduced possibility of causing neurotoxic side effects because of microtubule destabilization. This remains to be confirmed, but suggested a potential benefit for PP-13 anti-cancer activity, compared to taxanes and vinca-alkaloids that also affect interphase cells^16, 32^. It is worth noting that the only 1% of tubulin bound to PP-13 is sufficient to alter the microtubule dynamics essential for division. This could explain the effects observed in mitotic cells and the activation of the spindle assembly checkpoint. Thus, PP-13 is nontoxic for interphasic cells because of its 10- to 100-fold lower microtubule dynamics compared to that in mitotic cells^33^.

In addition, the PP-13 affinity for tubulin is much lower than that described for podophyllotoxin (Kb = 1.8 × 10^6^ M^−1^)^34^ and colchicine (Kb = 1.9 × 10^6^–1.2 × 10^7^ M^−1^)^35^. Such a lower affinity could be a favourable property as Kb has been shown to be correlated with tubulin ligand cytotoxicity^25^. This suggests that PP-13 could be better tolerated than colchicine and podophyllotoxin whose toxicities (mostly myelosuppression and neurotoxicity) hamper their use as anti-cancer drugs.

Moreover, the Kb of PP-13 is in a range fairly similar to those of other well-known colchicine binding site ligands, such as MTC (Kb = 4.7 × 10^5^ M^−1^)^36^ and nocodazole (Kb = 4 × 10^5^ M^−1^)^37^, indicating that PP-13 probably displays a sufficient affinity towards tubulin for its use as a promising antimitotic agent. Currently, a large number of tubulin colchicine site-binding agents are undergoing preclinical or clinical development^16, 22, 38, 39^. However, none of these agents display the same pyrrolopyrimidin scaffold as PP-13 that makes this compound an attractive candidate for further pre-clinical investigations.

It is widely known that solid malignancies are prone to develop acquired MDR after conventional chemotherapy treatment, mainly because of overexpression of transmembrane efflux pumps, such as MRP1 MRP2, BCRP and P-glycoprotein^40^. In this context, there is a keen interest to develop therapeutic alternatives with high cytotoxicity against MDR cancer cells. The recent FDA-approval of eribulin mesylate that overcome the MDR cell phenotype^41, 42^ illustrates this effort. In our study, PP-13 was shown to overcome efflux-mediated chemoresistance and could thus present a strong advantage over vinca-alkaloids that are actively effluxed by MRP1 and P-glycoprotein transporters or over taxanes that are exported by P-glycoprotein and MRP2^16^.

To confirm the PP-13 anticancer effects *in vivo* that were observed *in vitro*, we used the model of tumour xenografts in the chorioallantoic membrane of living chick embryos. Such *in ovo* experiments are accepted as potential alternatives to mammalian models for the *in vivo* assessment of drug toxicity and pharmacokinetics^43^, especially in the cancerology field, since tumour implantation is facilitated by CAM physiological immunodeficiency^44–46^. This model also allows the evaluation of tumour metastatic invasion owing to the intense vascularization of the egg^45^. We showed that PP-13 at a concentration of 0.17 µmol.L^−1^ effectively inhibits *in vivo* tumour growth with no detectable toxicity for the growing embryo. PP-13 also significantly reduced tumour cell dissemination *in vivo*, which suggested potential antimetastatic effects.

In summary, we described a pyrrolopyrimidine derivative (PP-13) that inhibited the growth of a wide range of cancer cell types, including targeted therapy-resistant and multidrug-resistant (MDR) cell lines. PP-13 caused prometaphase arrest associated with aberrant multipolar mitotic spindles, microtubule disorganization, and activation of spindle assembly checkpoint proteins. *In vitro* experiments revealed that PP-13 directly inhibits microtubule assembly by targeting the colchicine-binding site in β-tubulin. Finally, PP-13 exerted potent antitumour effects *in vivo* without noticeable toxicity in the chicken embryos. These findings suggest new prospects for PP-13 that could represent a promising alternative to conventional spindle poisons, such as taxanes or vinca-alkaloids in the case of MDR cancer cells.

## Methods

### Chemical reagents, antibodies and proteins

Molecule PP-13 (purity >95% by liquid chromatography – mass spectrometry) came from the Institut Curie (CNRS chemical library, 2004 version), was dissolved in anhydrous dimethyl sulfoxide (DMSO, Carl Roth, Karlsruhe, Germany) and stored in a 10 mmol.L^−1^ stock solution. Paclitaxel, colchicine, formaldehyde, and Hoechst 33342 were from Sigma-Aldrich (Saint Quentin Fallavier, France). The anti-α-tubulin and anti-actin antibodies were purchased from Santa Cruz (Clinisciences, Montrouge, France). The anti-γ-tubulin antibody was from Sigma. The anti-phospho-histone H3 and anti-cleaved caspase 3 (Asp^175^) antibodies were purchased from Cell Signaling (Ozyme, Saint Quentin Yvelines, France). Anti-centrin-2 antibodies were from Merck Millipore (Molsheim, France). Goat anti-mouse Alexa Fluor-488 and anti-rabbit Alexa Fluor-568 secondary antibodies were obtained from Life Technologies (Saint-Aubin, France). The tubulin used in the tubulin polymerization assay was isolated by Ecrins Therapeutics Services from bovine brain as previously described^47^, while the tubulin used in transmission electron microscopy (TEM) and the competition fluorescence assay was isolated from calf brains^48, 49^. The calf brains used to purify the tubulin were a kind donation of Ganadería Fernando Díaz. They come from 6 to 12 months old calfs sacrificed at the slaughterhouse Matadero Madrid-Norte S A Pol. Ind. Sur (Autovía Madrid-Burgos, 32,300, 28750 San Agustín del Guadalix, Madrid). These brains were extracted as other parts of the animals at the slaughterhouse following the commercial procedures. Since the brains used were officially approved as meat for human consumption, no further permissions from a comitee were required.

### Cell lines

NSCLC (H358, H322, A549, H1975, H1650, H3255, PC9 and H460), other human cancer cell lines from different organs (HCT116, HT29, MCF7, PC3, HeLa, colo829, A375, A7, NIH3T3, HEK293), human foetal lung fibroblast MRC5 cells, and human keratinocytes HaCat cells were obtained from the American Type Culture Collection (ATCC, Manassas, VA, USA), routinely tested and authenticated by the ATCC, and cultured as recommended. HeLa cells expressing mEGFP-α-tubulin and mRFP-H2B were a generous gift from Dr D. Gerlich^50^. HeLa cells expressing GFP-BubR1 were kindly provided by Pr A. Musacchio^51^. H358 cells stably expressing RFP-Histone H2B or cell lines overexpressing P-glycoprotein, MRP1, MRP2, or ABCG2 transporter were generated and selected in our laboratories. All cells were mycoplasma-free, cultured at 37 °C in the appropriate medium in a 5% CO2 humidified atmosphere and used within 6 months after thawing.

### Cell proliferation and apoptosis assays

Cell viability was analysed using the MTT assay as previously described^52^. Apoptotic cells with hypodiploid DNA staining were counted in “sub-G_1_” peaks using flow cytometry. Cleavage of caspase-3 was also detected by immunoblotting as previously described^53^.

### Cell-cycle analysis

Cells were harvested, fixed in 70% ethanol at 4 °C, incubated with anti-phospho-histone 3 Alexa Fluor 488-labelled antibodies diluted in 1% Foetal Bovine Serum (FBS)/0.25% Triton × 100/PBS for 1 h at room temperature, before staining with 20 µg.mL^−1^ propidium iodide in RNase A/PBS, as previously described^54^. The percentage of cells in the specific cell-cycle phases (G_0_/G_1_, S, G_2_, and M) was determined using an Accuri C6 flow cytometer (Becton Dickinson, Le Pont-de-Claix, France).

### Immunofluorescence analyses

Cells were grown on glass coverslips coated with 1 μg.mL^−1^ fibronectin, treated for 24 h, fixed and permeabilized with either 4% paraformaldehyde followed by 0.1% Triton × 100 incubation or with methanol at -20 °C. After washing and saturation with 5% BSA/PBS, cells were incubated for 1 h with primary antibodies (anti-α-tubulin, anti-γ-tubulin, anti-centrin-2 antibodies), washed twice again, and incubated with Alexa Fluor 488 and/or Alexa Fluor 568 secondary antibodies for 45 min at RT. DNA was stained with 20 µmol.L^−1^ Hoechst 33342, and coverslips were mounted with fluorescence mounting medium (DAKO, Trappes, France). Fluorescent images were captured using a multiphoton confocal laser-scanning microscope (LSM 510, Carl Zeiss, Jena, Germany) equipped with a Plan Apochromat 100×/1.4 NA oil objective and acquisition software LSM 510. All images are z-stacked.

### Time-lapse videomicroscopy

HeLa cells expressing alpha-tubulin-EGFP H2B-mRFP or GFP-BubR1 were seeded on 2-well LabTek slides (Nunc, D. Dutscher, Brumath, France) coated with 1 μg.mL^−1^ fibronectin and allowed to grow for 24 h prior to imaging. After treatment, the slide was placed on a 37 °C heated stage (at 5% CO_2_) and images were acquired every 3 min by a spinning disk confocal laser microscope (Andromeda iMIC, FEI, Munich, Germany) equipped with a Plan-Apochromat 40×/1.4 Oil objective and an EMCCD camera (iXon3897, Andor, Belfast, UK). For each time point, a stack of 15 planes (thickness: 2 µm) was recorded. Acquisition (LA) and off-line analysis (OA) software programs were used.

### Tubulin polymerization assay

The tubulin polymerization assay was performed by Ecrins Therapeutics Services (www.ecrins-therapeutics-services.com). Briefly, a half-area 96-well plate (Greiner Bio-one, Courtaboeuf, France) was charged with pure tubulin (40 µmol.L^−1^) in a PIPES-based polymerization buffer (80 mM K-PIPES pH 6.8, 1 mM MgCl_2_, 1 mM EGTA). After the addition of 1 mmol.L^−1^ GTP, tubulin assembly was followed at 37 °C in the presence of colchicine, paclitaxel or increasing concentrations of PP-13, by turbidimetry variation at 350 nm every 30sec during 1h (FLUOstar Omega, BMG Labtechnologies). The experiment was performed in triplicate. A negative control contained an equivalent quantity of DMSO as the sample with higher concentration of PP-13 (25 µmol.L^−1^). The solubility of the compound in the polymerization buffer was checked prior to the test.

### Transmission electron microscopy (TEM)

Tubulin assembly was performed in GAB buffer (3.4 mol.L^−1^ glycerol, 10 mmol.L^−1^ sodium phosphate (NaPi), 1 mmol.L^−1^ EGTA, 6 mmol.L^−1^ MgCl_2_, 1 mmol.L^−1^ GTP, pH 6.7) with 25 µmol.L^−1^ of pure tubulin and increasing amounts of PP-13. Samples were fixed with 0.1% glutaraldehyde. Subsequently, the reaction was stopped with 100 mmol.L^−1^ glycine, and then 4 µL of a 1/10 dilution in water of the sample was mounted into a carbon-coated 400 mesh copper grid. Samples were counterstained with 2% (w/v) uranyl acetate prior to examination using TEM. Micrographs were obtained using a JEOL 1230 transmission electron microscope at 100 KeV and a 16 megapixel TemCam-F416 camera from TVIPS.

### Competition fluorescence assay

The tubulin target site of PP-13 and its associated binding affinity constant (Kb) were determined by competitive assay using a fluorescent colchicine analogue MTC (2-methoxy-5-(2,3,4-trimethoxyphenyl)-2,4,6-cycloheptatrien-1-one) that reversibly binds to the colchicine-binding site^55, 56^. Equal amounts of pure tubulin and MTC (10 µmol.L^−1^) were mixed in a phosphate-based buffer (10 mmol.L^−1^ NaPi, 0.1 mmol.L^−1^ GTP). After addition of increasing concentrations of PP-13, the displacement of MTC binding to tubulin was measured by fluorescence changes using Fluoromax-2 (Jobin-Yvon SPEX, Longjumeau, France) (excitation wavelength: 350 nm, emission spectra: 372–525 nm). Tubulin and MTC alone served as negative controls for fluorescence normalization. Assuming 0.8 MTC sites per tubulin heterodimer and 4.7 × 10^5^ M^−1^ as the MTC binding constant^36^, the PP-13 binding affinity constant was evaluated using the Equigra v5 program^25^.

### *In vivo* experiments

A chick embryo tumour growth and metastasis assay (InOvotion, Grenoble, France) was performed as previously described^44, 57, 58^. According to the French legislation, no ethical approval is needed for scientific experimentations using oviparous embryos (decree n° 2013–118, February 1, 2013; art. R-214–88). Briefly, fertilized White Leghorn eggs (SFPA, Hendrix Genetics group, Saint Brieuc) were incubated at 38 °C with 60% relative humidity for 9 days. The chorioallantoic membrane (CAM) was then dropped, and a 1-cm^2^ window was cut in the eggshell above the CAM (at day 9). H358 cells were harvested by trypsinization, washed with complete medium, and suspended in serum-free medium. A 50 µL inoculatum of 1.10^6^ H358 cells was added directly onto the CAM of each egg. Eggs were randomly allocated into 3 groups of 17–19 eggs to get sufficient surviving embryos at the end of the experiments. At day 10, when tumours began to be detectable, eggs were treated every two days for 10 days by dropping 100 µL of either 50 µmol.L^−1^ paclitaxel, 0.17 µmol.L^−1^ PP-13 or 0.5% DMSO in PBS (vehicle) onto the tumour. The dropwise addition of a solution onto the large tumour area that depresses the CAM surface was found to be a suitable method, which avoided leakage and dispersion of the compounds. Then, the windows were sealed with cellophane tape and the eggs were returned to the incubator. At day 19, the upper portion of the CAM was removed and transferred to PBS, and the tumours were carefully cut away from normal CAM tissue and weighed. In parallel, a 1-cm^2^ portion of the lower CAM was collected to evaluate the number of H358 cells. To count these cells, genomic DNA was extracted, and qPCR analysis was performed by using primers specific for genomic human Alu/repetitive sequences^59^. Finally, the toxicity of the treatment was evaluated by scoring the number of dead embryos and looking for morphological or functional abnormalities in surviving embryos (Supplementary Fig. S12).

### Statistical analysis

Continuous variables were compared using the non-parametric Mann-Whitney *U*-test or Kruskal-Wallis test. Categorical variables were compared using Fisher’s exact test. All analyses were performed using Statview software. Statistical significance was defined as *p* values <0.05.

### Data availability

The datasets generated during and/or analyzed during the current study are available from the corresponding author on reasonable request.

## Electronic supplementary material

Supplementary information

Supplementary Figure S6

Supplementary Figure S7

Supplementary Figure S8

Supplementary Figure S9

## References

[1] Iams WT, Sosman JA, Chandra S (2017). Novel Targeted Therapies for Metastatic Melanoma. Cancer J. Sudbury Mass.

[2] Seeber A, Gastl G (2016). Targeted Therapy of Colorectal Cancer. Oncol. Res. Treat..

[3] Di Cosimo S, Baselga J (2010). Management of breast cancer with targeted agents: importance of heterogeneity. [corrected]. Nat. Rev. Clin. Oncol..

[4] Crafton SM, Salani R (2016). Beyond Chemotherapy: An Overview and Review of Targeted Therapy in Cervical Cancer. Clin. Ther..

[5] Dholaria B, Hammond W, Shreders A, Lou Y (2016). Emerging therapeutic agents for lung cancer. J. Hematol. Oncol.J Hematol Oncol.

[6] Maione P (2015). Overcoming resistance to targeted therapies in NSCLC: current approaches and clinical application. Ther. Adv. Med. Oncol..

[7] Wang, L., Wang, H., Song, D., Xu, M. & Liebmen, M. New strategies for targeting drug combinations to overcome mutation-driven drug resistance. *Semin. Cancer Biol*., doi:10.1016/j.semcancer.2016.11.002. 10.1016/j.semcancer.2016.11.002 27840276

[8] Olaussen KA, Postel-Vinay S (2016). Predictors of chemotherapy efficacy in non-small-cell lung cancer: a challenging landscape. Ann. Oncol. Off. J. Eur. Soc. Med. Oncol..

[9] Curtis, S. A., Cohen, J. V. & Kluger, H. M. Evolving Immunotherapy Approaches for Renal Cell Carcinoma. *Curr. Oncol. Rep*. **18**, (2016). 10.1007/s11912-016-0542-9 27475806

[10] Khanna P, Blais N, Gaudreau P-O, Corrales-Rodriguez L (2017). Immunotherapy Comes of Age in Lung Cancer. Clin. Lung Cancer.

[11] Margolin, K. The Promise of Molecularly Targeted and Immunotherapy for Advanced Melanoma. *Curr. Treat. Options Oncol*. **17**, (2016). 10.1007/s11864-016-0421-5 27461037

[12] Zhao X, Subramanian S (2017). Intrinsic Resistance of Solid Tumors to Immune Checkpoint Blockade Therapy. Cancer Res..

[13] Ferrara R (2016). Tubulin inhibitors in non-small cell lung cancer: looking back and forward. Expert Opin. Pharmacother..

[14] Hurbin A (2005). Cooperation of amphiregulin and insulin-like growth factor-1 inhibits Bax- and Bad-mediated apoptosis via a protein kinase C-dependent pathway in non-small cell lung cancer cells. J. Biol. Chem..

[15] Hurbin A, Dubrez L, Coll J-L, Favrot MC (2003). Inhibition of apoptosis by amphiregulin via an insulin-like growth factor-1 receptor-dependent pathway in non-small cell lung cancer cell lines. Ann. N. Y. Acad. Sci..

[16] Dumontet C, Jordan MA (2010). Microtubule-binding agents: a dynamic field of cancer therapeutics. Nat. Rev. Drug Discov..

[17] Prudent R (2012). Pharmacological inhibition of LIM kinase stabilizes microtubules and inhibits neoplastic growth. Cancer Res..

[18] Singh P, Rathinasamy K, Mohan R, Panda D (2008). Microtubule assembly dynamics: an attractive target for anticancer drugs. IUBMB Life.

[19] Meraldi P (2016). Centrosomes in spindle organization and chromosome segregation: a mechanistic view. Chromosome Res. Int. J. Mol. Supramol. Evol. Asp. Chromosome Biol..

[20] Maiato H, Logarinho E (2014). Mitotic spindle multipolarity without centrosome amplification. Nat. Cell Biol..

[21] Alfieri C (2016). Molecular basis of APC/C regulation by the spindle assembly checkpoint. Nature.

[22] Lu Y, Chen J, Xiao M, Li W, Miller DD (2012). An Overview of Tubulin Inhibitors That Interact with the Colchicine Binding Site. Pharm. Res..

[23] Andreu JM, Gorbunoff MJ, Lee JC, Timasheff SN (1984). Interaction of tubulin with bifunctional colchicine analogues: an equilibrium study. Biochemistry (Mosc.).

[24] Bane S, Puett D, Macdonald TL, Williams RC (1984). Binding to tubulin of the colchicine analog 2-methoxy-5-(2′, 3′, 4′-trimethoxyphenyl)tropone. Thermodynamic and kinetic aspects. J. Biol. Chem..

[25] Díaz JF, Buey RM (2007). Characterizing ligand-microtubule binding by competition methods. Methods Mol. Med..

[26] Apoptosis in the absence of caspase 3. *Publ. Online 04 Oct. 2001 Doi101038sjonc1204815* **20**, (2001).

[27] Meek, D. W. The role of p53 in the response to mitotic spindle damage. *Pathol. Biol. (Paris)* **48**, 246–254 (2000). 10858957

[28] Wang Y, Jin F, Higgins R, McKnight K (2014). The current view for the silencing of the spindle assembly checkpoint. Cell Cycle.

[29] Matson DR, Stukenberg PT (2011). Spindle Poisons and Cell Fate: A Tale of Two Pathways. Mol. Interv..

[30] Gascoigne KE, Taylor SS (2008). Cancer cells display profound intra- and interline variation following prolonged exposure to antimitotic drugs. Cancer Cell.

[31] Shi J, Mitchison TJ (2017). Cell death response to anti-mitotic drug treatment in cell culture, mouse tumor model and the clinic. Endocr. Relat. Cancer.

[32] Ogden A, Rida PCG, Reid MD, Aneja R (2014). Interphase microtubules: chief casualties in the war on cancer?. Drug Discov. Today.

[33] Yvon AM, Wadsworth P, Jordan MA (1999). Taxol suppresses dynamics of individual microtubules in living human tumor cells. Mol. Biol. Cell.

[34] Cortese F, Bhattacharyya B, Wolff J (1977). Podophyllotoxin as a probe for the colchicine binding site of tubulin. J. Biol. Chem..

[35] Fernando Díaz J, Andreu JM (1991). Kinetics of dissociation of the tubulin-colchicine complex. Complete reaction scheme and comparison to thermodynamic measurements. J. Biol. Chem..

[36] Medrano FJ, Andreu JM, Gorbunoff MJ, Timasheff SN (1991). Roles of ring C oxygens in the binding of colchicine to tubulin. Biochemistry (Mosc.).

[37] Xu K, Schwarz PM, Ludueña RF (2002). Interaction of nocodazole with tubulin isotypes. Drug Dev. Res..

[38] Wang Y (2016). Structures of a diverse set of colchicine binding site inhibitors in complex with tubulin provide a rationale for drug discovery. FEBS J..

[39] Massarotti A, Coluccia A, Silvestri R, Sorba G, Brancale A (2012). The Tubulin Colchicine Domain: a Molecular Modeling Perspective. ChemMedChem.

[40] Chen Z (2016). Mammalian drug efflux transporters of the ATP binding cassette (ABC) family in multidrug resistance: A review of the past decade. Cancer Lett..

[41] Osgood, C. L. *et al*. FDA Approval Summary: Eribulin for Patients with Unresectable or Metastatic Liposarcoma who have Received a Prior Anthracycline-Containing Regimen. *Clin. Cancer Res. Off. J. Am. Assoc. Cancer Res*., doi:10.1158/1078-0432.CCR-16-2422 (2017). 10.1158/1078-0432.CCR-16-2422 PMC10182892 28242632

[42] Aseyev O, Ribeiro JM, Cardoso F (2016). Review on the clinical use of eribulin mesylate for the treatment of breast cancer. Expert Opin. Pharmacother..

[43] Vargas A, Zeisser-Labouèbe M, Lange N, Gurny R, Delie F (2007). The chick embryo and its chorioallantoic membrane (CAM) for the *in vivo* evaluation of drug delivery systems. Adv. Drug Deliv. Rev..

[44] Prudent R (2013). Azaindole derivatives are inhibitors of microtubule dynamics, with anti-cancer and anti-angiogenic activities: Anti-angiogenic inhibitors of microtubule dynamics. Br. J. Pharmacol..

[45] Ribatti D (2016). The chick embryo chorioallantoic membrane (CAM) assay. Reprod. Toxicol. Elmsford N.

[46] Li, M. *et al*. The In Ovo Chick Chorioallantoic Membrane (CAM) Assay as an Efficient Xenograft Model of Hepatocellular Carcinoma. *J. Vis. Exp. JoVE*., doi:10.3791/52411 (2015). 10.3791/52411 PMC4692648 26484588

[47] Castoldi M, Popov AV (2003). Purification of brain tubulin through two cycles of polymerization-depolymerization in a high-molarity buffer. Protein Expr. Purif..

[48] Andreu JM (2007). Large scale purification of brain tubulin with the modified Weisenberg procedure. Methods Mol. Med..

[49] Díaz JF, Andreu JM (1993). Assembly of purified GDP-tubulin into microtubules induced by taxol and taxotere: reversibility, ligand stoichiometry, and competition. Biochemistry (Mosc.).

[50] Steigemann P (2009). Aurora B-mediated abscission checkpoint protects against tetraploidization. Cell.

[51] Overlack K (2015). A molecular basis for the differential roles of Bub1 and BubR1 in the spindle assembly checkpoint. eLife.

[52] Jeannot V (2016). Synergistic activity of vorinostat combined with gefitinib but not with sorafenib in mutant KRAS human non-small cell lung cancers and hepatocarcinoma. OncoTargets Ther..

[53] Busser B (2010). Amphiregulin promotes BAX inhibition and resistance to gefitinib in non-small-cell lung cancers. Mol. Ther. J. Am. Soc. Gene Ther..

[54] Vignon C (2013). Flow cytometric quantification of all phases of the cell cycle and apoptosis in a two-color fluorescence plot. PloS One.

[55] Alejandre-García I (2015). Cytotoxic Activity and Chemical Composition of the Root Extract from the Mexican Species Linum scabrellum: Mechanism of Action of the Active Compound 6-Methoxypodophyllotoxin. Evid.-Based Complement. Altern. Med. ECAM.

[56] La Regina G (2007). Arylthioindole inhibitors of tubulin polymerization. 3. Biological evaluation, structure-activity relationships and molecular modeling studies. J. Med. Chem..

[57] El Hasasna H (2016). Rhus coriaria suppresses angiogenesis, metastasis and tumor growth of breast cancer through inhibition of STAT3, NFκB and nitric oxide pathways. Sci. Rep..

[58] Mikaelian I (2013). Genetic and pharmacologic inhibition of mTORC1 promotes EMT by a TGF-β-independent mechanism. Cancer Res..

[59] Zijlstra A (2002). A quantitative analysis of rate-limiting steps in the metastatic cascade using human-specific real-time polymerase chain reaction. Cancer Res..

[60] Jordan MA, Toso RJ, Thrower D, Wilson L (1993). Mechanism of mitotic block and inhibition of cell proliferation by taxol at low concentrations. Proc. Natl. Acad. Sci. USA.

