# Supplementary material for: Identification of pyrrolopyrimidine derivative PP-13 as a novel microtubule-destabilizing agent with promising anticancer properties
Source: Sci Rep. 2017 Aug 31;7:10209. doi: 10.1038/s41598-017-09491-9 (PMC5579042; doi:10.1038/s41598-017-09491-9)
Supplement: Supplementary file 1 — Supplementary information [file 41598_2017_9491_MOESM1_ESM.pdf]

Identification of pyrrolopyrimidine derivative PP-13 as a novel microtubule-destabilizing agent with promising anticancer properties

Pauline Gilson[1,2], Fernando Josa-Prado[3], Claire Beauvineau[4], Delphine Naud-Martin[4], Laetitia Vanwonterghem[1], Florence Mahuteau-Betzer[4], Alexis Moreno[5], Pierre Falson[5], Laurence Lafanechère[1], Véronique Frachet[1,6], Jean-Luc Coll[1], Jose Fernando Díaz[3], Amandine Hurbin[1*$], Benoit Busser[1,2$]

Supplementary information

Supplementary figure S1

Genotypic features of NSCLC cell lines:

Amino acid mutation is indicated. WT: wild-type, EGFR-TKI: EGFR tyrosine kinase inhibitor, p.?: not defined.

gene / cell line	TP53	KRAS	EGFR	others
A549	WT	Mutant G12S	WT	
H460	WT	Mutant Q61H	WT	
H1650	Mutant p.?	WT	Mutant delE746_A750 (exon 19)	PTEN loss confering resistance to EGFR-TKI
H1975	Mutant R273H	WT	Mutant: L858R (exon 21), T790M (exon 20) confering resistance to EGFR-TKI	
H358	P53 deleted	Mutant G12C	WT	
PC9	Mutant R248Q	WT	Mutant delE746–A750 (exon 19)	
H3255	Mutant p.?	WT	Mutant L858R (exon 21)	
H322	Mutant R248L	WT	WT	

Supplementary figure S2

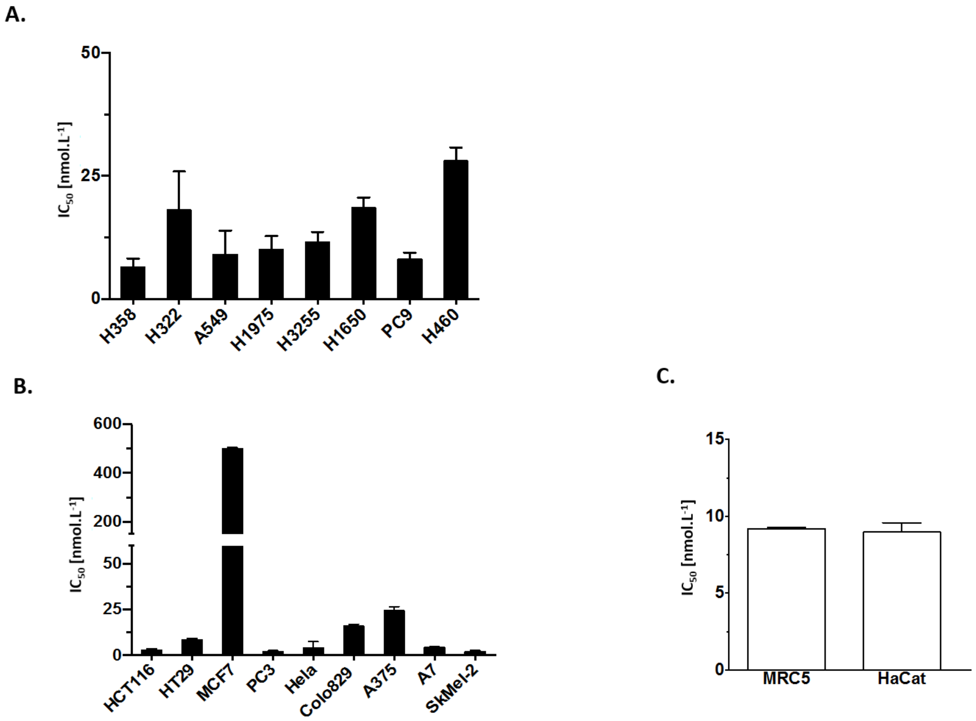

Paclitaxel inhibited the proliferation of human cancer cell lines.

Paclitaxel concentrations required to inhibit cell growth by 50% (IC_{50}) at 72 h were determined by MTT assays in NSCLC cells (A.), in other representative cancer cell lines from various origins (B.), and in human fetal lung fibroblast MRC5 cells and in human keratinocyte HaCat cells (C.). Data represent the mean ± SD of three independent experiments (in $nmol.L^{-1}$).

Supplementary figure S3

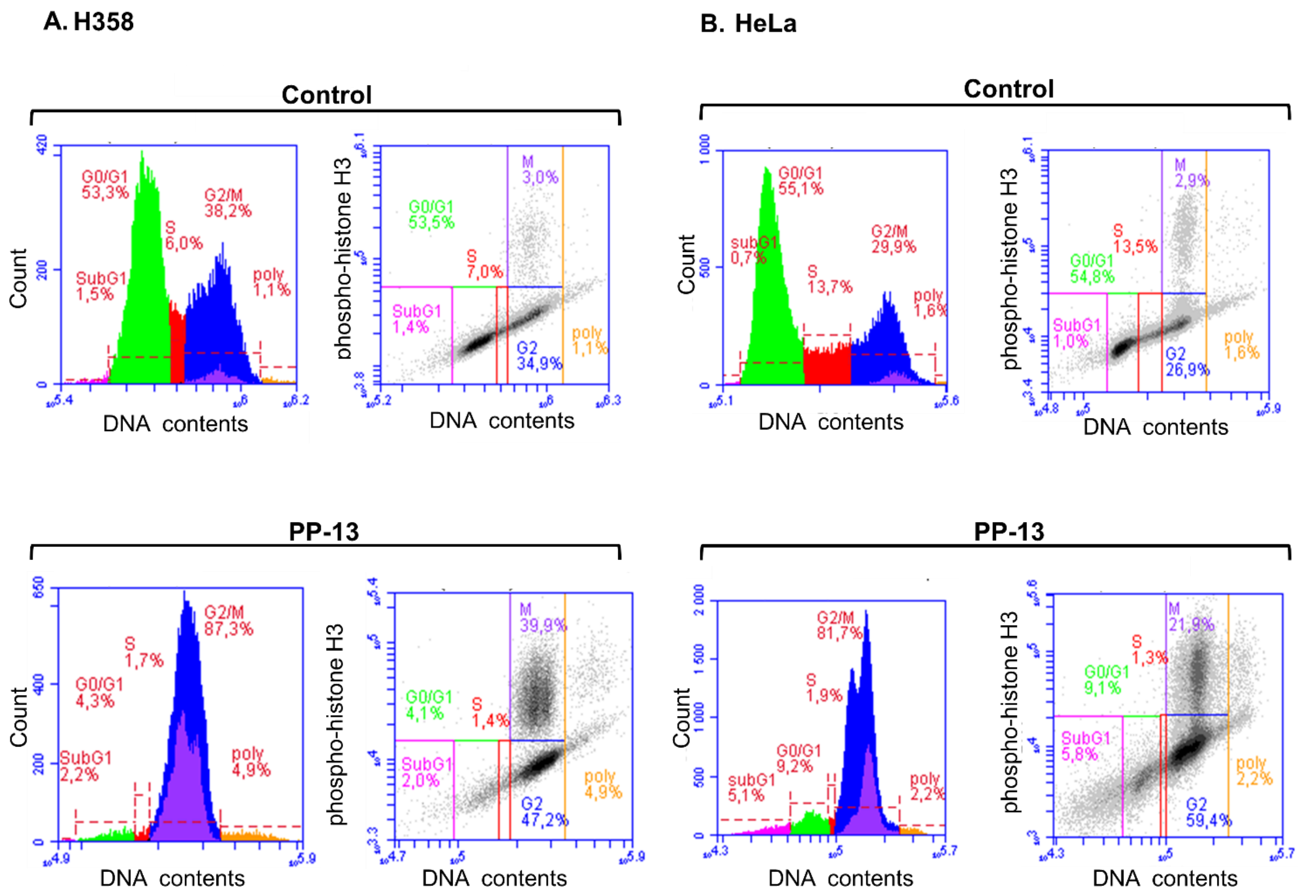

PP-13 interferes with mitotic progression of H358 and HeLa cells. H358 (**A.**) and HeLa (**B.**) cells were treated with PP-13 at 170 or 120 nmol.L^{-1} respectively for 18 h. The percentage of cells in each phase of the cell-cycle was determined by flow cytometry. Representative histograms of ≥ 3 independent experiments show the repartition of cells in the different cell-cycle phases considering the DNA content. Associated dot plots show cell distribution according to phospho-histone H3 signal and DNA contents. Phosphorylated-histone H3 is used as a late G2/mitosis-specific marker. Propidium iodide estimates the cell DNA content.

Supplementary figure S4

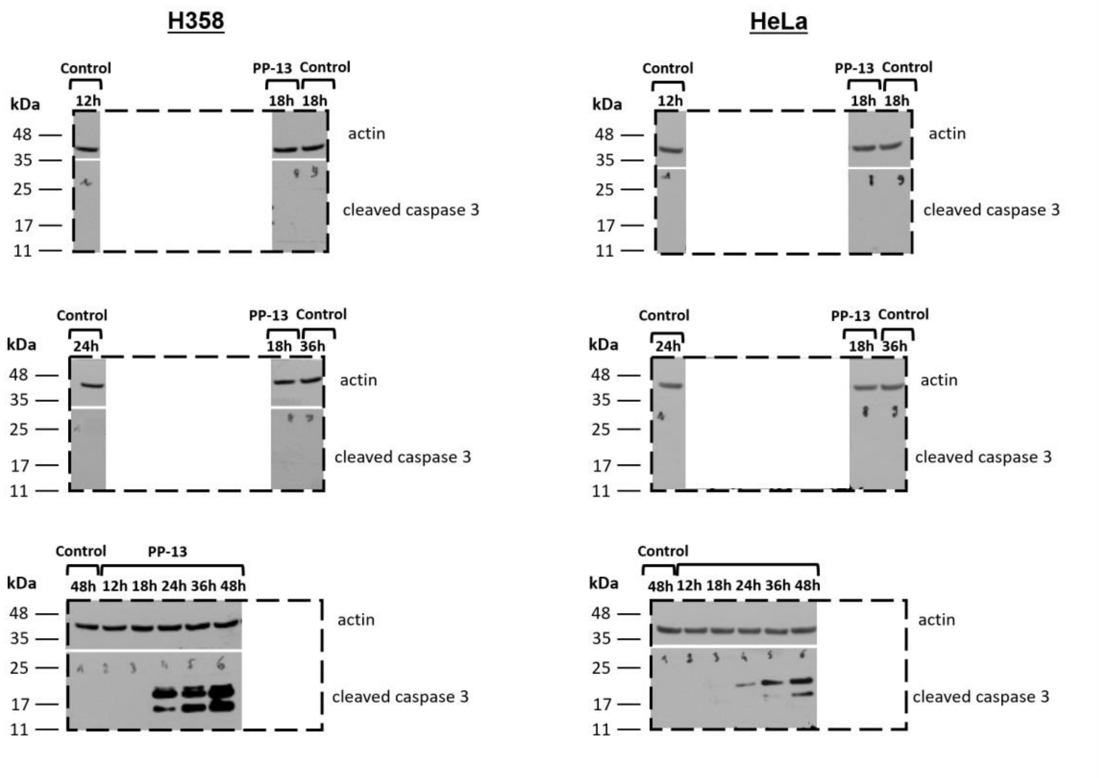

PP-13 strongly induced the cleavage of caspase-3 in both H358 and HeLa cells.

Cleavage of caspase 3 was evaluated by western blotting. Actin was used as a loading control. Full-length blots are presented. Only conditions presented in results are shown.

Supplementary figure S5

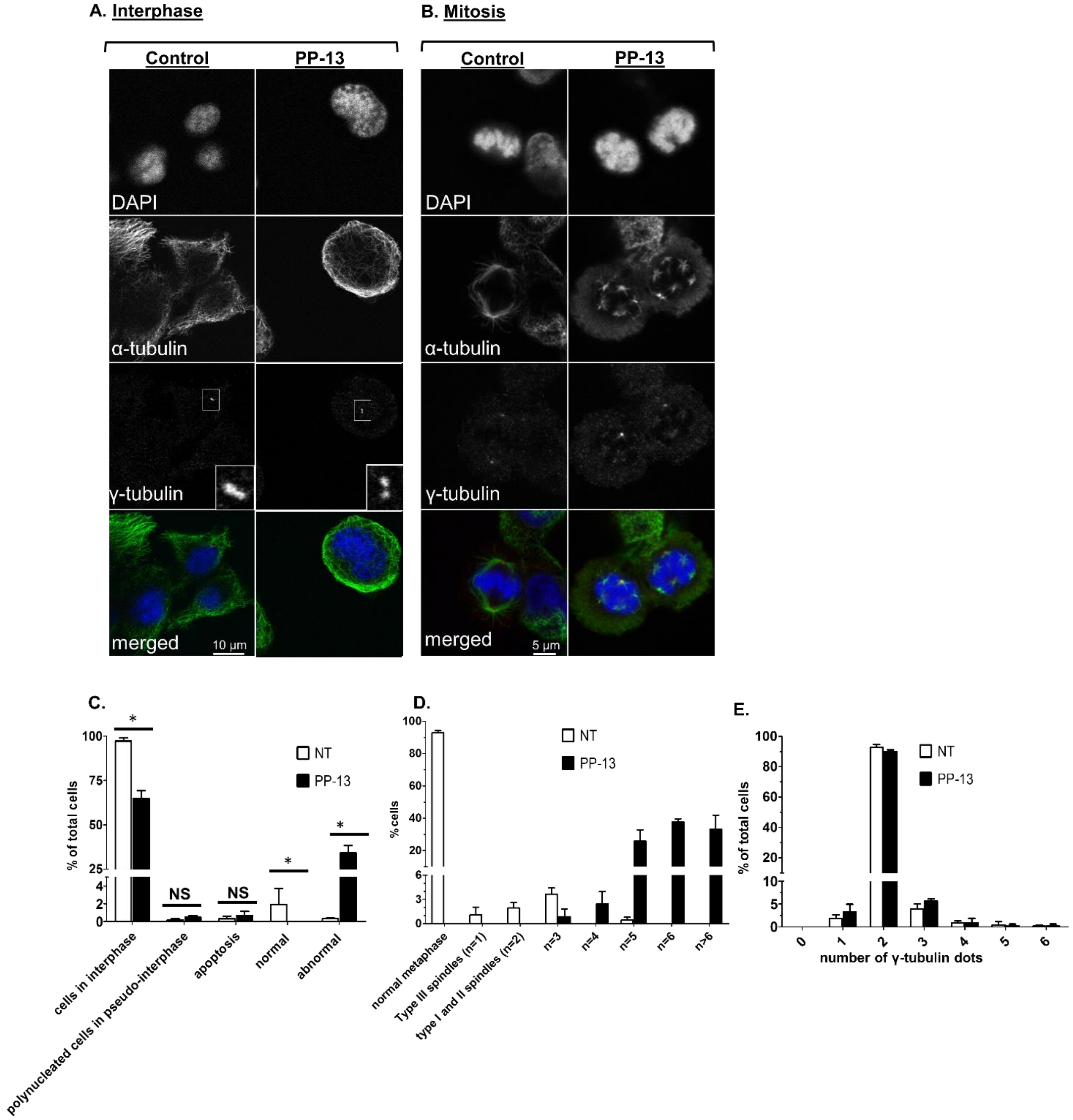

PP-13 interferes with mitotic spindle architecture and centrosome formation in H358 cells. H358 cells were treated or not with 170 nmol.L^{-1} PP-13 for 24h. **A-B.** Representative confocal microscopy images of microtubules and centrosomes in cells in interphase (**A.**) or in mitosis (**B.**). In green: α-tubulin, in red: γ-tubulin, in blue: DNA staining with DAPI. Insets represent a zoom of the boxed area. **C.** Quantification of interphase, mitotic, apoptotic and polynucleated cells. Results for 300 cells randomly chosen and assigned. *, $p<0.05$. NS, not significant. **D-E.** Quantification of mitotic spindles (**D.**) and γ-tubulin dots (**E.**) per mitotic cell. At least 100 mitotic cells were randomly chosen and scored in each condition. Mitotic spindles were classified according to a well-established nomenclature (Jordan et al., 1993): type I and II spindles are bipolar (n=2) with at least one uncongressed chromosomes, type III spindles are monopolar (n=1), and multipolar spindles present more than two poles (n >2). n: number of mitotic spindles.

Supplementary Figure S6

The cell division process in control cells.

The cell division process of non-treated α-tubulin-EGFP H2B-RFP HeLa cells was followed over time with time-lapse videomicroscopy. Time-lapse interval was set to 3 minutes. Control cells undergoing mitosis exhibit typical features of the different cell-cycle phases: interphase, prophase, metaphase, anaphase I, anaphase II, cytokinesis, interphase. In red: H2B-RFP, in green: α-tubulin-EGFP.

Supplementary Figure S7

Following cell prometaphase arrest, a fraction of PP-13-treated cells undergo mitotic catastrophe. α-tubulin-EGFP H2B-RFP HeLa cells were treated with 120 $nmol.L^{-1}$ PP-13, and cells were followed over time with time-lapse videomicroscopy. Time-lapse interval was set to 3 minutes. Under PP-13 treatment, some cells directly undergo apoptosis following mitotic blockade. In red: H2B-RFP, in green: α-tubulin-EGFP.

Supplementary Figure S8

Following cell prometaphase arrest, a fraction of PP-13-treated cells undergo asymmetric division. α-tubulin-EGFP H2B-RFP HeLa cells were treated with 120 $nmol.L^{-1}$ PP-13, and cells were followed over time with time-lapse videomicroscopy. Time-lapse interval was set to 3 minutes. Under PP-13 treatment, the fate of mitotically-arrested cells was various. Some cells initiated aberrant cytokinesis with asymmetric division leading to the generation of non-viable polynucleated cells. In red: H2B-RFP, in green: α-tubulin-EGFP.

Supplementary Figure S9

Under PP-13 treatment, generated polyploid cells undergo apoptosis or moved forward into mitosis. α-tubulin-EGFP H2B-RFP HeLa cells were treated with 120 $nmol.L^{-1}$ PP-13, and cells were followed over time with time-lapse videomicroscopy. Time-lapse interval was set to 3 minutes. In some cases, polynucleated cells generated from asymmetric division can initiate a second cell division process before undergoing apopotosis. In red: H2B-RFP, in green: α-tubulin-EGFP.

Supplementary figure S10

A.

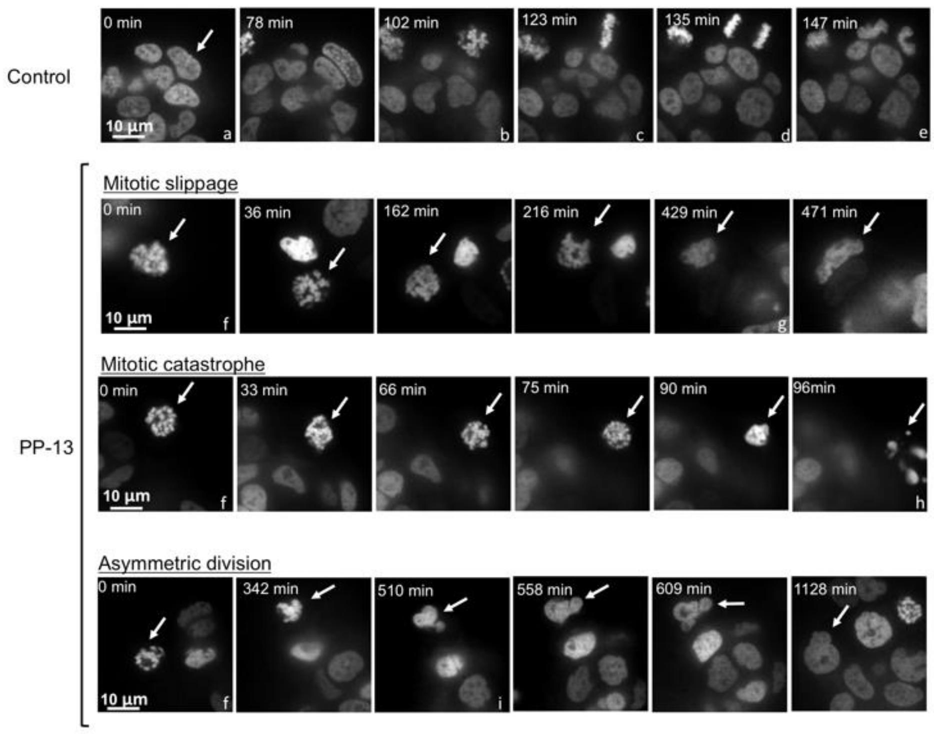

B.

**Death in mitosis**	12 %
**Asymmetric division**	24 %
**Mitotic slippage**	64 %

PP-13 blocks H358 cells in prometaphase following mitotic slippage or direct apoptotic death. H2B-RFP H358 cells were treated or not with 170 nmol.L-1. PP-13 and cell division process was followed over the time using time-lapse videomicroscopy. **A.** Representative pictures from time-lapse videomicroscopy experiment are shown. In white: H2B-RFP. Control cells undergoing mitosis exhibit typical features of the different cell-cycle phases: a. interphase. b. prophase. c. metaphase. d. anaphase II; e. interphase. PP-13 induces cell prometaphase arrest (f), then mitotic slippage (g), direct apoptotic death (h) or asymmetric division (i). White arrows show the cell of interest. Time is indicated on each picture (in minutes). **B.** Percentages of different cell fate induced by PP-13 treatment were estimated by time-lapse videomicroscopy, after following 25 treated H2B-RFP H358 cells over time.

Supplementary figure S11

A. PP-13 (1 µmol.L^{-1}) **B. PP-13 (10 µmol.L^{-1})**

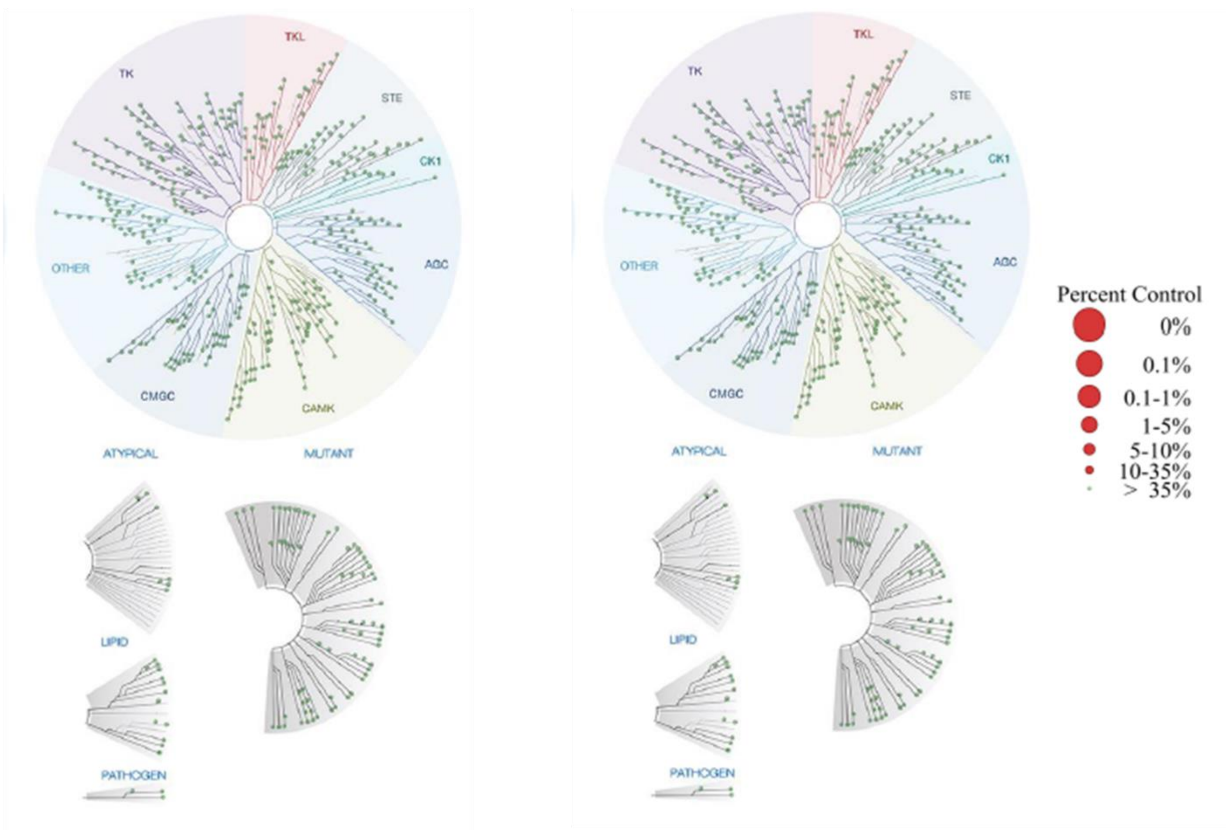

PP-13 is not a kinase inhibitor. Interactions between PP-13 and more than 450 human kinases and disease relevant mutant variants were evaluated by a kinome array (KINOME*scan* screening platform, LeadHunter™ Discovery Services, DiscoverX Corp., Fremont, United States), as previously described (Fabian et al., Nature Biotechnology 2005). PP-13 was screened at 1 (**A.**) and 10 µmol.L^{-1} (**B.**). Red dots show significant kinase inhibition. The percentage represent the resultant kinases activity in presence of PP-13 compared to the negative control.

Supplementary figure S12: PP-13 does not induce noticeable toxicity in chicken embryos.

A. Number of dead and surviving embryo for the different experimental groups after 10 days of treatments

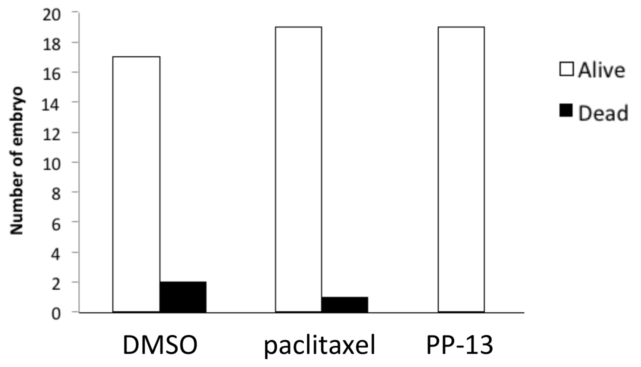

B. Twenty-two checkpoints were probed on chicken embryos to evaluate toxicity:

Analyzed part of the embryos	checkpoints
Head	size, closure, eyes, ear, face and branchial arc derivatives, mobility
Body	size, axis deformation, ventral and dorsal closures, caudal formation, sexual area
Limbs	size, axis morphology, mobility
Skin	appendage formation, attachement, blood vessel
Extra-embryonic structures	vascularization, transparency, attachment, blood vessel

C. Number of abnormality observed on surviving embryos after 10 days of treatment:

	Head	Body	limbs	Skin	Extra Emb.
DMSO	0	0	0	0	0
Paclitaxel	0	0	0	0	0
PP-13	0	0	0	0	0

0 = no abnormality
